# Dynamic sparse x-ray nanotomography reveals ionomer hydration mechanism in polymer electrolyte fuel-cell catalyst

**DOI:** 10.1126/sciadv.adp3346

**Published:** 2024-10-09

**Authors:** Zirui Gao, Christian Appel, Mirko Holler, Katharina Jeschonek, Kai Brunnengräber, Bastian J. M. Etzold, Michal Kronenberg, Marco Stampanoni, Johannes Ihli, Manuel Guizar-Sicairos

**Affiliations:** ^1^ Paul Scherrer Institut, 5232 Villigen PSI, Switzerland.; ^2^ ETH and University of Zürich, 8092 Zürich, Switzerland.; ^3^ Brookhaven National Laboratory, Upton, NY 11973-5000, USA.; ^4^ Technical University of Darmstadt, 64287 Darmstadt, Germany.; ^5^ Friedrich-Alexander-Universität Erlangen-Nürnberg, 90762 Fürth, Germany.; ^6^ Carl Zeiss SMT, 73447 Oberkochen, Germany.; ^7^ University of Oxford, Oxford OX1 2JD, UK.; ^8^ École Polytechnique Fédérale de Lausanne, 1015 Lausanne, Switzerland.

## Abstract

Tomographic imaging of time-evolving samples is a challenging yet important task for various research fields. At the nanoscale, current approaches face limitations of measurement speed or resolution due to lengthy acquisitions. We developed a dynamic nanotomography technique based on sparse dynamic imaging and 4D tomography modeling. We demonstrated the technique, using ptychographic x-ray computed tomography as its imaging modality, on resolving the in situ hydration process of polymer electrolyte fuel cell (PEFC) catalyst. The technique provides a 40-time increase in temporal resolution compared to conventional approaches, yielding 28 nm half-period spatial and 12 min temporal resolution. The results allow a quantitative characterization of the water intake process inside PEFC catalysts with nanoscale resolution, which is crucial for understanding their electrochemical mechanisms and optimizing their performance. Our technique enables high-speed operando nanotomography studies and paves the way for wider application of dynamic tomography at the nanoscale.

## Teaser

Sparse dynamic nanotomography method provides 40-time speed increase and sub–30 nm resolution for in situ hydration of PEFC.

## INTRODUCTION

Studying dynamic systems with computed tomography (CT) has been of great interest ever since the technique’s first introduction, as its penetrative and nondestructive properties provide a unique ability to image the interior of operando systems. The development of CT techniques able to image dynamic processes across a wide range of lengthscales and timescales has thus been an important topic over the past decades, and has seen applications in various research fields such as biology, chemistry, or energy materials (*1*–*9*). These techniques have been used in different imaging modalities for a diverse spectrum of applications, ranging from CT imaging of cardiac and respiratory motions (*2*, *10*, *11*), x-ray microtomography of wing beats of insects (*12*), or electron nanotomography of molecular interactions (*13*).

The performance of dynamic tomography techniques is often defined by their achievable spatial and temporal resolution. While these performance measures vary greatly across different sample sizes and application scenarios, a common constraint experienced by almost all these techniques is the limitation on measurement speed. This includes limitations on both the highest achievable temporal resolution, and the total acquisition time required in case of periodic processes. For the fastest applications of x-ray microtomography, state-of-the-art methods can achieve a speed of 1000 tomograms per second (*7*). However, for nanotomography the imaging rates are considerably slower, with a timescale normally on the order of hours (*14*, *15*).

This limitation is chiefly driven by the underlying assumption that the sample is static during tomographic acquisition. To measure a three-dimensional (3D) volume at a desired resolution, a certain number of tomographic projections need to be taken at different relative orientation angles between the object and the incident illumination. The number of projections is determined by the Crowther sampling criterion and, for a fixed volume, it grows inversely proportional to the sought resolution (*16*). When aiming for higher resolution, the increased number of projections increases concomitantly the acquisition time. Another limiting factor is the mechanical overhead needed to rotate the sample, which can become a bottleneck in high-speed applications (*17*).

Furthermore, for a given photon flux, the total acquisition time for a 3D volume scales inversely proportional to the fourth power of the sought spatial resolution (*18*), resulting in substantially longer acquisition times when aiming to resolve nanoscale features. So while nanoscale dynamics are of paramount importance for many applications, the acquisition times put strong limits on either the achievable temporal resolution, or the measurable sample volume for dynamic tomography.

To address these challenges, several 4D CT methods have been proposed on the basis of various methodological approaches. Some of these methods focus on improved hardware design and acquisition protocol to increase acquisition speed (*7*, *17*). Others try to reduce the required number of projections so that the same dynamic process can be imaged with fewer measurements, which are often referred to as sparse tomography (*19*–*24*).

For sparse tomography, the number of projections is reduced to only a subset of measurements compared to the Crowther criterion, by reducing the angular sampling (*20*, *23*). Applying sparsity in the measurement largely enhances measurement speed, yet inevitably impairs imaging quality compared to the conventional approach due to angular undersampling. However, these effects can often be mitigated with specially designed reconstruction methods, based on models of the properties of the sample and its dynamics. Existing techniques use different strategies, such as prior information about the sample (*25*) or numerical constraints in the modeling of dynamics (*26*).

We here propose a measurement approach and reconstruction algorithm designed specifically for the challenges of the nanotomography regime. Our method achieves an unprecedented 40-fold increase in temporal resolution, while maintaining the same level of spatial resolution and sample volume attainable as in regular nanotomography. Such speed increase is enabled by a combination of sparse sampling and 4D tomography modeling, which leverages correlations along the time dimension. While most existing dynamic tomography methods reconstruct a temporal series of tomograms for the dynamic process, our approach uses step function–based 4D model to decompose hundreds of temporal frames into three independent tomograms, two for sample density (initial and final state) and one for transition time. This approach largely reduces the number of unknowns in the 4D tomography model and allows reconstruction from sparsely sampled datasets without need of prior knowledge of the sample and is not affected by ambiguities or blurring introduced by numerical constraints during tomogram reconstruction. We further incorporated a nonrigid CT (NCT) approach into the tomogram reconstruction (*27*). This technique allows us to account for any potential sample deformations that may occur during the dynamic tomography acquisition, resulting in a more robust tomogram reconstruction with enhanced spatial resolution and quantitative accuracy.

As a first demonstration, we imaged the controlled hydration process of a catalyst layer inside the membrane electrode assembly (MEA) of a hydrogen polymer electrolyte fuel cell (PEFC). PEFCs hold great potential as environmentally friendly alternatives to combustion-based engines for the transportation sector, since they use H_2_ and O_2_ to generate water and electric energy. Besides notable progress in research on the material, PEFCs still need to increase in energy efficiency and to reduce the use of costly noble metals to be an important pillar of a sustainable energy sector (*3*, *22*, *28*). Chemically modified and new designs for catalytic sites with improved activity, at reduced amount of noble metals, are setting new standards for the catalyst’s performance potential. However, when these materials are tested under realistic conditions, i.e., in catalyst layers within fuel cell stacks, it becomes apparent that other factors limit the PEFC performance (*29*). Insufficient water management is a particular example, where liquid water saturates the complex structure of the catalyst layer and hinders the transport of gaseous components, namely, H_2_ and O_2_, to the catalytically active sites. On the contrary, liquid water can be needed to ionically contact active sites which are not contacted through the ionomer. While characterization of water content in operating fuel cells is already accessible via neutron imaging (*30*) and full field x-ray tomography on the micrometer scale (*31*), quantitative measures at the nanoscale are still missing. Since the electrochemical reaction takes place on the nanoscale, it is crucial to obtain the catalyst’s layer structure on this length scale and as close as possible to realistic working conditions. Of particular interest at the nanoscale is the ionomer in the catalyst, which is only able to fulfil its proton-conducting role after it absorbs water molecules. We here demonstrate an unprecedented combination of 28 nm half-period spatial resolution in 3D with a temporal resolution of 12 min using ptychographic x-ray CT (PXCT), to investigate the humidity-induced changes in a catalyst layer sample with a 20 μm diameter and 10 μm height.

**Fig. 1.**
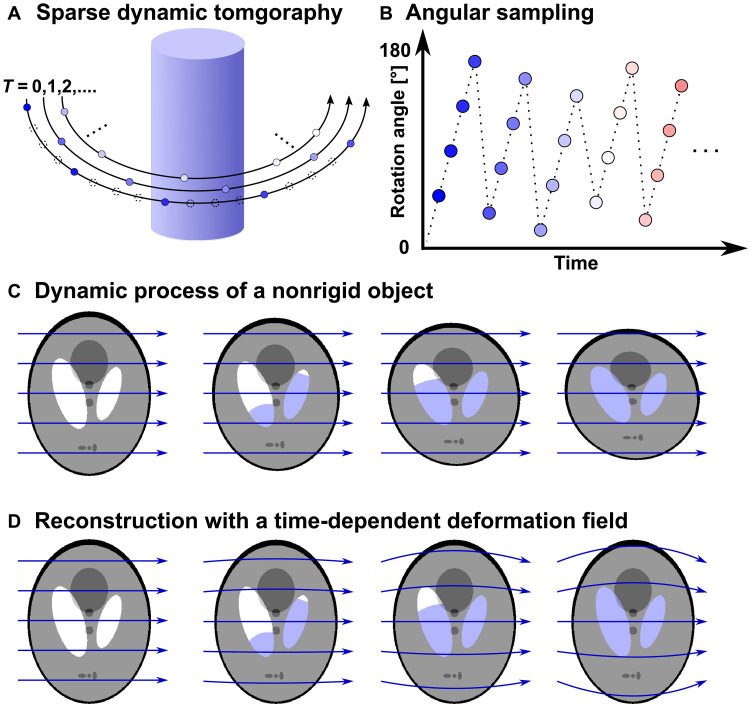
Schematic of the sparse dynamic tomography technique. (**A**) Measurement strategy of sparse dynamic tomography, each dot represents one projection measured at a given sample orientation. Dashed-line dots represent projections that would be needed by the Crowther criterion but that are skipped by sparse sampling. (**B**) Plot of tomography rotation angles versus time. At the start of each sparse tomogram, i.e., time frame, an angular offset is added based on golden ratio. (**C**) Schematic of a simulated water filling process measured while the object is deforming during the dynamic process. (**D**) Reconstruction of the dynamic process with deformation field correction, which accounts for the deformation of the sample and allows localized dynamics to be retrieved on a stable sample structure. Blue arrows in (C) represent normal projection lines, and curved lines in (D) represent curved integration path for projections using the nonrigid computed tomography (NCT) approach.

The sparse dynamic nanotomography method consists of two parts: measurement and reconstruction. The measurement technique, as illustrated in Fig. 1A, includes a set of sparse sequential tomography measurements, i.e., time frames, covering the time lapse of the whole dynamic process. Similar to conventional tomography, in these measurements, projections of the sample are measured at different sample orientation angles from 0° to 180°. Sparsity is applied to each tomography rotation by taking only a small proportion of the total number of rotation angles required by the Crowther criterion. An angular offset, calculated with golden ratio, was added to the starting angle of each tomogram to maximize diversity of information content for more efficient spatial sampling, as shown in Fig. 1B (*19*, *21*, *24*).

**Fig. 2.**
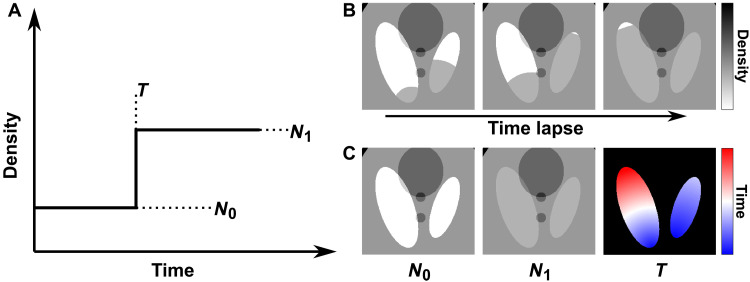
Step function model for dynamic reconstruction. (**A**) Step function model of dynamic process in one voxel. The step function is uniquely defined by values *N*_0_, *N*_1_, and *T*. (**B**) Selected time frames that represent the simulated dynamic liquid filling process. (**C**) Variables *N*_0_, *N*_1_, and *T* that model the process. The colormap for *T* represents transition time, ranging from the start to the end of the process, with colors from blue to red.

In our reconstruction approach, we model the dynamic processes combining two methods, as shown in Fig. 1D. First, we consider deformations of the sample that cause relative movement, expansion, or contraction of the whole or any part of the sample. This is defined as any change of the sample that can be mapped to the starting state by a time-dependent deformation-vector field (*27*). Second, we represent the local changes in electron density (ED) by a voxel-wise temporal step function, as described in Fig. 2A. It assumes that the local changes at each voxel occur within one of the tomographic time frames. This assumption is naturally derived from dynamic processes composed of step-like transitions, such as the simulated liquid filling shown in Fig. 2B. However, we demonstrated that its extended application based on spatial upsampling can also be used to model gradual transitions and discussed the performance of the reconstruction method under this scenario in Materials and Methods subsection “Numerical simulations.”

The combination of these two models, shown in Fig. 1D, can accurately account for various dynamic processes, for example, phase transitions such as solid melting, liquid condensation, or evaporation, which include concomitant deformation of the sample structure (*32*, *33*), chemical reactions such as oxidation or lithiation in energy materials, quantifying the dynamics and water content for cement hydration (*34*), and mechanical deformation including crack formation.

An iterative temporal refinement technique was used for tomography reconstruction. All measured projections are first aligned with the tomography projection alignment method described in (*35*). Then, we apply the NCT method to reconstruct deformations of the sample during the whole measurement process. As previously described in (*27*), this method extracts a vector-based time-dependent deformation field, which is then used on the tomographic projections and back-projections. The NCT method can effectively model and correct the deformations of the sample during measurement and allows us to use a simplified model for the local change of density in individual voxels. Using the nonrigid correction, we can assume that the changes are “localized,” such that local ED changes in the sample can be represented at their starting position throughout the whole process. This simplifies the local dynamics greatly, as it allows us to exclude any intertwining effects between neighboring voxels caused by deformation or movement, and to therefore use a much simpler model to encode the remainder dynamics. A more detailed description and results of the NCT method can be found in Materials and Methods.

In our approach, we assume that the imaged quantity of each voxel changes like a step function in time, as shown in Fig. 2, which shows a transition from the start value *N*_0_ to the end value *N*_1_ at a certain time point *T*. Dynamics that consist of one step function for each voxel can be uniquely defined by the three variables *N*_0_, *N*_1_, and *T*, shown in Fig. 2C. These three variables can be interpreted as the initial state, the final state, and the transition time of each voxel and can be used to reconstruct a full 3D volume for each time interval. This is illustrated in Fig. 2 (B and C) for a simulated dataset of a liquid filling process.

The reconstruction problem is then reduced to reconstructing *N*_0_, *N*_1_, and *T* variables for each voxel of the 3D volume. We developed a method based on iterative refinement, where we use adapted projections and back-projections according to the reconstructed deformation field. Refinements are applied simultaneously to the *N*_0_, *N*_1_, and *T* variables at each voxel based on differences between the projections generated by the current iteration of the variables and the measured projections. As a result, the method is able to reconstruct the start and end states of the sample at full spatial resolution and retrieve the transition time values with a temporal resolution equal to the length of a time frame, i.e., the measurement time of one sparsely sampled tomogram. It should be emphasized that the method reconstructs simultaneously all three variables, and it is not needed to acquire a static measurement neither at the beginning, nor at the end, of the dynamics. A more detailed description of the reconstruction algorithm can be found in Materials and Methods. Note that the transition time *T* can be used to directly extract contours or isosurfaces of a phase transition or liquid filling. Then, these reduced volumetric parameters can be considered one step ahead in the analysis of the 4D dataset.

During the reconstruction, we do not apply any spatial constraint nor any restriction on the values of *N*_0_, *N*_1_, nor on the difference between them. Instead, we only assume one transition between two states for each voxel, and the local values can either increase, decrease, or stay unchanged. This also means that apart from capturing water condensation in empty pore space, i.e., an increase in ED, the method also allows reconstruction of other structural changes, such as crack formation or a decrease in density accompanied by a volume expansion or swelling process, as it is demonstrated in the experimental results.

**Fig. 3.**
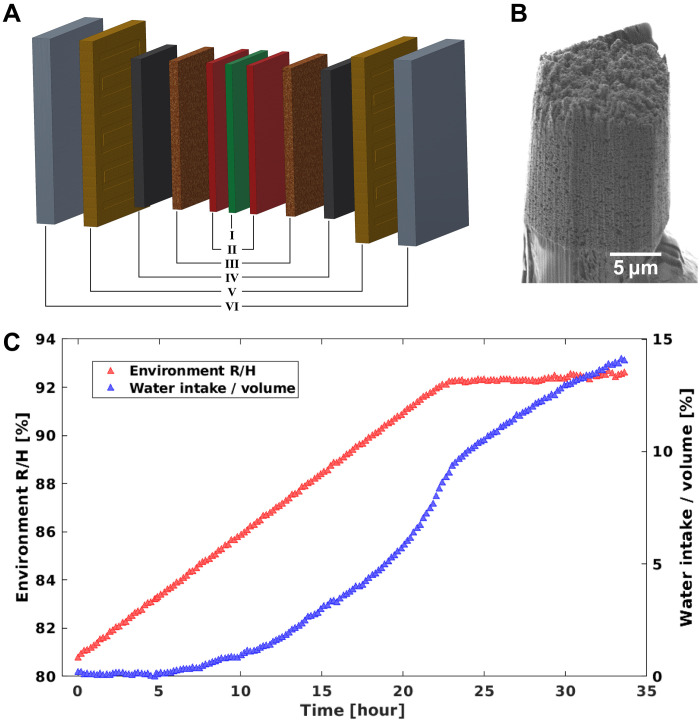
PEFC sample. (**A**) Schematic of the structure of a PEFC. The cell consists of (I) polymer electrolyte membrane layer composed of Nafion, (II) catalyst layers, (III) microporous layers, (IV) gas diffusion layers, (V) flow channels, and (VI) bipolar plates. (**B**) SEM image of the sample pillar, extracted from the catalyst layer (II). (**C**) Measurement process of the controller hydration of PEFC catalyst sample. Red triangles show the environment RH versus time, each triangle represents the starting time of one sparsely sampled tomography measurement. Blue triangles show the amount of water intake, which is estimated from the integrated ED obtained from 2D projections.

We demonstrated our sparse dynamic nanotomography method by imaging the vapor condensation and structural response upon water intake in a standard platinum/carbon (Pt/C) catalyst layer inside a PEFC. A schematic representation of a PEFC stack is shown in Fig. 3A. At the heart of the PEFC stack, we find a polymer electrolyte membrane composed of Nafion (I). Two catalyst layers (II), cathode and anode, surround the membrane and host the electrochemical reactions, hydrogen oxidation, and oxygen reduction. The two catalyst layers are sandwiched by two microporous (III) and two gas diffusion layers (IV), which guarantee sufficient transport of gaseous components, H_2_ and O_2_, to and liquid water from the catalyst layers. Together, these layers (I to IV) form the MEA. The full PEFC stack is complemented by two flow channels (V) for providing H_2_, O_2_, and H_2_O and two bipolar plates (VI).

Inside the catalyst layers (II), the electrochemical reactions are driven by catalytic active sites of small hydrophilic Pt nanoparticles with an average diameter of 3 nm. The nanoparticles are distributed on the surface of a hydrophobic carbon support, Vulcan XC72R, and partially covered by the ionomer. The ionomer acts not only as a binder but also as the proton conducting element between the anode and cathode catalyst layers. It can only fulfil this role in its hydrated state, which also explains why PEFCs are usually operated at high relative humidity (RH) of 80 to 100%. However, upon water intake, the nanoscale structure of the ionomer is expected to change with a decrease in density and an expansion into unoccupied space (*36*). These structural changes may additionally hinder the transport of the gaseous components within the catalyst layers together with liquid water condensation, which increasingly highlights the importance of understanding the role of water in this complex environment.

We prepared a 20-μm-diameter pillar extracted from a catalyst coated membrane for a MEA of a PEFC. A scanning electron microscope (SEM) image of the sample is shown in Fig. 3B. Experiments were performed at room temperature while the atmospheric conditions surrounding the sample were controlled by an airflow system, the latter providing humidified nitrogen with a RH ranging between 80 and 93%. As shown in Fig. 3C, the RH was slowly increased from 81 to 92% in steps of 0.1% over a time span of 34 hours. The sample was then kept at a RH reading of 92%, which is the saturation value of the humidity sensor. The sample continues to absorb water after the 92% humidity reading is reached, as shown in the water intake in Fig. 3C, which is calculated from the integrated ED of the sample, calculated from the 2D projections.

For each time interval, indicated in Fig. 3C, a tomography measurement is made with sparsely sampled angular orientations. Each of these sparse tomograms contain 25 sample angular orientations in the range from 0° to 180°, which corresponds to a sparsity ratio of 2.2% compared to the 1122 projections required by the Crowther criterion for a 28 nm half-period resolution. As mentioned above, the starting angles at each time frame were adjusted using a golden ratio approach (*19*, *24*, *37*), allowing more efficient spatial sampling. Each sparse tomography measurement took 12 min, including overhead, and the RH was increased by 0.1% for each sparse tomography measurement.

## RESULTS

**Fig. 4.**
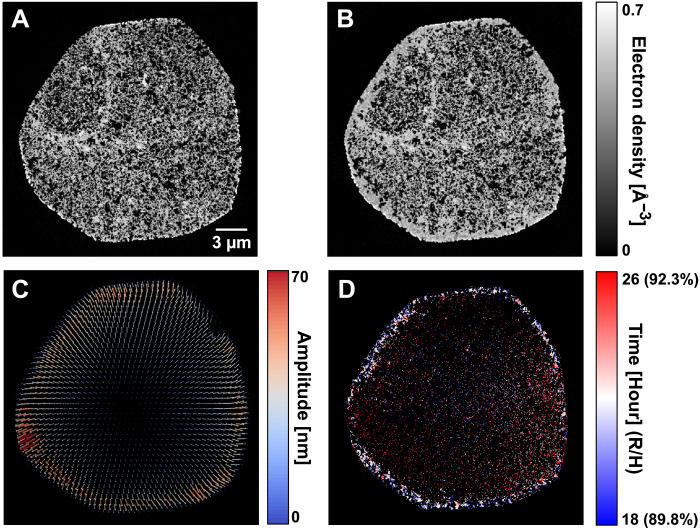
Dynamic tomography reconstruction result of the catalyst sample. (**A**) Axial tomographic slice of the reconstructed ED tomogram at start state. (**B**) The same slice of ED tomogram at end state, where filling of the pores can be observed. (**C**) Reconstructed deformation vector field of the sample between the start and end state. Arrows indicating the vector field are scaled up 15 times to improve visibility. (**D**) Map of the transition time *T*, or corresponding environment RH in parentheses, of the same slice. This map shows with color coding the time point when localized changes occur in each voxel.

Using the NCT method, we obtained a deformation vector field that characterizes the expansion of the sample, as shown in Fig. 4C. After the nonrigid correction, we reconstruct the *N*_0_, *N*_1_, and *T* variables that define the dynamic process, shown in Fig. 4 (A, B, and D, respectively). A comparison of the improvement obtained by using the NCT method, and more details on the reconstruction procedure, can be found in Materials and Methods.

**Fig. 5.**
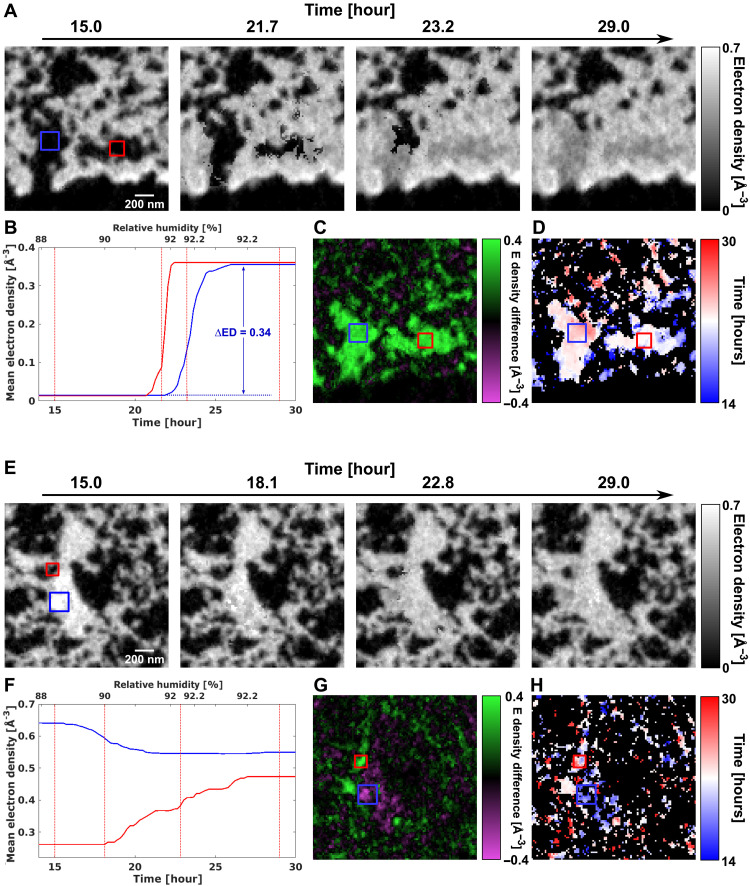
Reconstruction inset of 1.7 × 1.7 μm^2^ areas in an axial tomography slice. (**A**) Reconstructed time-lapse ED tomograms showing pore condensation. (**B**) Evolution over time of the average ED of the regions enclosed by blue and red rectangles in (A). Vertical lines denote the time frames of the tomogram insets shown in (A). The increase in ED matches that of water, 0.333 e/Å^3^. (**C**) ED difference between the start and end states. (**D**) Map of transition time of the region. (**E**) to (**H**) show the same contents, but for a second region that demonstrates water absorption and swelling of the ionomer.

During the controlled hydration process, we observe water condensation in the outer layer of the sample pillar. This is shown in Fig. 4 (A and B), which depict axial tomography slices from the reconstructed tomograms of the start and the end states of the sample. We can clearly see the difference in ED between these states at low and high RH, especially the filling of pores in the outer layer. The transition time *T* shown in Fig. 5D can further be correlated with the readout of environment RH during the dynamic measurement.

Using the variables *N*_0_, *N*_1_, and *T*, we can retrieve the tomograms at each time frame during the whole dynamic hydration process. To identify the actual changes in ED and to illustrate the high resolution of our method, we zoom in to examine two 1.7 × 1.7 μm^2^ regions inside the sample, as shown in Fig. 5. In the first region (Fig. 5, A to D), we can clearly observe the process of water condensation into the porous catalyst structure over the timescale of a few hours. Initial changes in the ED become visible after the RH approaches 91%. Focusing on the two highlighted regions (red and blue boxes), we observe that these changes take place within about 5 hours. In this time interval, the average ED in the highlighted region in Fig. 5A increases by approximately 0.34 e/Å^3^, as shown in Fig. 5B, a value that matches the tabulated ED of water of 0.333 e/Å^3^. We further note that our method is able to capture multiple stages of the condensation process, since a total of 25 time frames are measured for this time span of 5 hours, and some of the representative frames are shown in Fig. 5A.

The ED difference between the final and initial state is shown in Fig. 5C. The difference clearly shows that the porous regions of the catalyst become filled with water. The water condensation in the larger pores occurs in two steps, as shown in the transition time *T* in Fig. 5D. Initially, some condensation is visible already early on at the edge of the larger pores, followed by a rather quick filling of the full volume. This agrees with the expected behavior of the catalyst layer. Water will initially not adsorb on the hydrophobic carbon surface, but on the small hydrophilic Pt nanoparticles, as well as get absorbed in the ionomer of the catalyst. Once condensation starts, it continues to draw water from the humid atmosphere and starts to fill up the pores rapidly.

The second region (Fig. 5, E to H) exhibits an entirely different behavior. During the increase of humidity, the ED in the central part of the ionomer decreases (blue box) from 0.64 to 0.55 e/Å^3^ and increases in the adjacent pore volume (red box) from 0.26 to 0.47 e/Å^3^. The quantitative change in ED, in particular the decrease in the ionomer, cannot be explained by water condensation but is instead related to structural changes within the ionomer. The ionomer is expected to change its nanoscale structure upon water intake. A notable decrease in density of up to 25% has been observed in literature (*36*), accompanied by swelling on a molecular level, which is below the resolution of our measurements. The fully wetted ionomer is expected to have an ED of 0.434 e/Å^3^ compared to 0.593 e/Å^3^ in its dry state. With a half-period resolution of 28 nm, we cannot resolve the high-density Pt nanoparticles, which are of approximately 3 nm in diameter and have an ED of ~5.167 e/Å^3^. Because of partial volume effects, the ED measured in each voxel is potentially higher due to the presence of these unresolved particles. In addition, each voxel may also contain the carbon support, with an ED of 0.59 to 0.61 e/Å^3^ that does not change with hydration. Nevertheless, we still observe a clear decrease in ED for multiple voxels within the second region, as highlighted in Fig. 5G, from which we can deduce which voxels are mostly occupied by the ionomer. Our method is also able to resolve the ionomer swelling into the surrounding pore space in 3D which is, to the best of our knowledge, not yet reported in the literature before at this resolution.

The substantial changes of the ionomer’s nanostructure demonstrate that water saturation and absorption in the PEFC catalyst layer plays not only a role in empty pores but can also induce crucial changes in the nanostructure of the ionomer. Optimizing the ionomer content is an important parameter that correlates with PEFC performance (*38*, *39*). Its swelling may further hinder the diffusion of gaseous components to the catalytic active sites, in particular when taking into account that in operando conditions additional water is generated as a product of the electrochemical reaction. The capability to directly image the ionomer swelling in situ will play an important role to study its structural response upon water intake from nanometer to micrometer length scales, and our method is ideally suited to capture these changes.

## DISCUSSION

We have introduced an experimental and reconstruction method for dynamic sparse x-ray nanotomography and demonstrated it by imaging the controlled hydration process of a PEFC catalyst. During the hydration process, this sample experienced a combination of representative changes, including deformation, pore filling, and ionomer swelling. We have observed in the nanoscale the wetting, including local expansion and decrease in ED, of one of its central components: the hydrophilic ionomer. Our findings offer crucial insights toward identifying the performance bottleneck of PEFC catalysts and enhancing their efficiency in the future.

The reconstructions, with a 3D half-period spatial resolution of 28 nm, were obtained from a dataset that was measured with 1/40 of the conventionally required number of projections. This corresponds to one 3D temporal frame every 12 min, which is a marked improvement over the conventionally needed 8 hours. Measurement time is a severe bottleneck for 3D imaging of nanoscale dynamics in representative volumes, our demonstration here reaches a rate of almost 362,000 resolution elements per second, which improves nanotomography imaging rates at this resolution by almost two orders of magnitude. Given that synchrotron experimental time is scarce and valuable, our development opens the door to a whole new regime of possible dynamical studies.

Furthermore, the implementation of the NCT correction allows our method to have excellent stability against sample movement or deformation, which enables identification and quantification of dynamic events with higher precision. The step function model also allows the transition time to be directly reconstructed instead of being obtained from postprocessing of tomograms, and this greatly eases the analysis steps in many dynamic studies where the key information is given by the time of transition.

Thanks to the versatility and robustness of our method, it can be applied to various length scales and illumination probes with minimal changes to the hardware and measurement protocols. As examples, these include x-ray microtomography, transmission electron tomography, and optical tomography. The technique can be readily generalized, for example, by modeling dynamics using a sequence of step transitions, as opposed to just one. In this manner, the method could be applied to more complex or periodic systems, such as operando imaging of the charge-discharge cycle of batteries. With the emerging upgrade to fourth generation synchrotrons, and concomitant improvement of optics and instrumentation, an increase of two orders of magnitude in the available coherent flux is expected. With this increase, the speed of the method can be brought down to seconds for nanoscale characterization, granting great potential for elucidating mechanisms of biological or chemical systems.

## MATERIALS AND METHODS

### Sample preparation

The catalyst sample was taken from a MEA for a polymer-electrolyte fuel cell. The membrane was produced by spray coating a commercial Nafion (NR-211, Ionpower) with a catalyst ink using a custom-built coating system. The catalyst ink was prepared by dispersing 20 mg of catalyst powder (HiSPEC 3000, Johnson Matthey) in a mixture of 138 μl of deionized water with less than 1.1 S cm^−1^ and 4841 μl of 2-propanol (99.9% VLS grade, Roth) with a vial tweeter. Nafion resin solution (20 wt %; EW 1100, Sigma-Aldrich) was added to the mixture to achieve an ionomer/carbon weight ratio of 0.54. The custom-built setup comprises a computerized numerical control system equipped with an ultrasonic spraying nozzle, for which argon is used as the carrier gas. The catalyst-coated membrane was prepared by spraying 160 cycles at a flow of 60 μl/min over an area of 2.5 × 2.5 mm^2^ of the Nafion membrane, which is placed on a heated plate beneath an infrared lamp to accelerate the drying process.

The imaged sample pillar was extracted from a mechanically cutout of a PEFC membrane, mounted on an SEM stub, using a focused Ga ion beam (Ga ions accelerated with 30 keV voltage) milling system. Milling was performed inside a SEM (Zeiss NVision 40 Gallium FIB/SEM). To minimize specimen beam damage, a weak beam of Ga ions (40 pA) was used to identify the area to be cut and to regularly inspect the milling progress. As a first step, a 13-nA Ga beam with an incidence angle perpendicular to the membrane surface was used to cut a set of parallel trenches, 25 to 30 μm in depth, into the membrane. Trenches were cut to extract a square shaped pillar with edge length of about 50 μm. Then, the edges of the square pillar were cleaned with a 3-nA Ga beam, and the pillar was transferred to an OMNY pin, a type of copper sample pin designed for nanotomography imaging (*40*), using a liftout procedure with the help of a micromanipulator. After being mounted on the OMNY pin, the sample pillar was further reduced in diameter to roughly 20 μm with a 1.5-nA Ga beam angled perpendicular to the top surface of the pillar. For fine-shaping of the pillar, the Ga beam was tilted to an incidence angle of 54° with respect to the top surface and hit the pillar from its side, while the pillar was rotated around its axis in steps of 15°. This way, a sample pillar with clear edges and nearly constant diameter of 20 μm from top to bottom can be prepared. An SEM image of the final sample pillar is shown in Fig. 3B. To note, a Ga beam intensity of 1.5 nA was used in the second cutting step to minimize damage to the ionomer structures in the sample. It was not further reduced to avoid lengthy cutting time and therefore minimize heat transfer to the specimen.

On the basis of the initial-state ED tomogram, in particular the ED variations as a function of distance to the pillar center, we concluded that the described FIB milling sample preparation procedure damaged the outermost 100 to 200 nm of the 20-μm-wide pillar, as seen in Fig. 4A. No systematic and radially symmetric ED variations, indicative of Ga deposition and sample preparation–associated damage, could be identified deeper into sample. Ga penetration/depth to this level is a common occurrence, with damaged areas being insignificant in volume compared to the whole sample, and typically has minimal impact on the results of scientific data analysis.

### Measurement and data preprocessing

The sparse dynamic nanotomography measurements were performed at the cSAXS beamline, Swiss Light Source, Paul Scherrer Institute, Switzerland. An x-ray energy of 6.2 keV was selected using a double-crystal Si (111) monochromator. A set of slits, located 22 m upstream of the sample, were set to a horizontal aperture of 20 μm, which creates a secondary source that coherently illuminates a Fresnel zone plate downstream with 200 μm diameter and 60 nm outermost zone width. The Fresnel zone plate was designed with locally displaced zones to improve imaging quality and phase accuracy (*41*). The sample was placed 1.48 mm downstream the focal point of the zone plate to get an illumination of 5 μm diameter on the sample. Coherent diffraction patterns were acquired using an in-vacuum Eiger 1.5M area detector (*42*) placed 5.23 m downstream of the sample inside a flight tube under vacuum. Ptychograms were measured using the flexible tomography nano-imaging end-station flOMNI (*43*), a dedicated instrument for x-ray scanning microscopy, which achieves positioning accuracy better than 10 nm by using laser interferometry feedback (*44*). The 2D projection field of view was 27 × 10 μm^2^. 2D ptychograms were measured following a Fermat spiral trajectory (*45*) with an average step size of 1 μm and a 0.05 s exposure time per point. Fast positioning for ptychography scan is achieved by combined motion of the sample and focusing zone plate (*46*), and each 2D scan took 23 s. For this experiment, we have modified the setup to be able to measure under controlled RH via an air flow system that mixes dry and humidified nitrogen gas, combined with a humidity sensor that monitors and controls the RH of the air surrounding the sample.

Before the dynamic tomography measurement, several groups of 2D scans were measured at different RH to locate the ionomer-rich regions in the catalyst and to estimate the amount of water intake. Before ramping up the RH, we waited 10 hours for the sample to stabilize at 81% RH, as shown in fig. S1A. We define the stabilized time point, at which the dynamic measurements of interest started, as *t*_0_ = 0, as shown in fig. S1A. All measurements before that time were excluded from the presented analysis.

As described in the main text and in Fig. 3C, sparsely sampled tomograms were measured repeatedly while the RH was gradually increased from 81 to 92% in 35 hours.

The sparse tomography measurement strategy is shown in fig. S1B. For each sparsely sampled tomogram, the sample was rotated to 25 different angles from 0° to 180°, and one ptychographic 2D projection was taken at each angle. After each tomogram, the sample was rotated back to 0°, and the process was repeated with an angular offset given by the golden ratio (*37*). In total, 173 sparsely sampled tomograms were measured, and for simplicity, we define the starting time of each sparsely sampled tomogram as *t* = *t*_0_, *t*_1_, …*t*_171_, *t*_172_ and use Proj(*t_i_*) to denote the set of 25 projections measured between *t_i_* and *t*_*i*+1_.

Ptychography scans were reconstructed with an iterative phase retrieval algorithm, with of 300 iterations of difference map (*47*) followed by 500 iterations of maximum likelihood (*48*), using the PtychoShelves package (*49*). Then, from the reconstructed complex-valued images, we extract the phase component and use phase unwrapping (*50*) to remove constant and linear phase offset terms (*51*). We denote the outcome phase projections as *P*(*t_i_*) = phase{Proj(*t_i_*)} for further analysis.

All projections were subsequently pre-aligned with a tomography alignment approach based on multiresolution projection matching with deep subpixel accuracy (*35*). The volume percentage of water intake at each time frame, shown in Fig. 3C and fig. S1A, was estimated from the 2D projections as

$$W_{i}=\frac{A}{\rho V_{p}N_{A}Z}\eta_{\text{ED}}\left[\sum P\left(t_{i}\right)-\sum P\left(t_{0}\right)\right]$$ (1)

where *A* = 18 g/mol is the molecular mass of water, ρ = 1 g/cm^3^ is the density of water, *V*_p_ is the volume of the sample pillar, *N*_A_ is the Avogadro constant, and *Z* = 10 is the number of electrons in a water molecule. The coefficient η_ED_, used for converting phase into ED (*52*), is given by

$$\eta_{\text{ED}}=\frac{1}{\lambda {\mathrm{lr}}_{0}}$$ (2)

where λ is the x-ray wavelength, *l* is the side length of the pixel in the projection, and *r*_0_ is the classical electron radius.

### Nonrigid computed tomography

In micro- and nanotomography applications, deformations of the sample often have a notable effect on the measurement of dynamic processes and, in some cases, can become the limiting factor for imaging quality or resolution (*53*). In our case, as deformations of the sample structure were observed during its water intake process, we used the NCT method (*22*) to quantitatively reconstruct the deformation field and account for these deformations explicitly. By doing this, the dynamic changes per each voxel can be more accurately described by the step function model described in Fig. 2.

A deformation vector field $\Gamma \left(\overset{⃑}{r},t\right)$ describes the deformation of the sample structure at time *t* relative to its starting state at *t*_0_, with the latter used as the reference state. At any time point *t_i_*, projections of the reconstructed model, considering the deformation field, can be calculated as

$$\hat{P}\left(t_{i}\right)=A_{N}\left\{\Gamma \left(\overset{⃑}{r},t_{i}\right)\right\}N\left(\overset{⃑}{r},t_{i}\right)$$ (3)

where $N\left(\overset{⃑}{r},t_{i}\right)$ is the reconstructed sample at *t_i_*, and $A_{N}\left\{\Gamma \left(\overset{⃑}{r},t_{i}\right)\right\}$ is the projection matrix under curved geometry given by deformation field $\Gamma \left(\overset{⃑}{r},t_{i}\right)$ , as defined in equation 3a in (*27*).

In the NCT method (*27*), the time-evolving deformation vector field $\Gamma \left(\overset{⃑}{r},t\right)$ is calculated from discretized vector fields $\Gamma \left(\overset{⃑}{r},t_{i}\right)$ , which describe the deformation of the *i*th tomogram. However, in our case, a large number of tomograms were measured with a very low sparsity ratio, making it unfeasible to reconstruct one discretized vector field for each tomogram. Therefore, we approximate the dynamics of the deformation field as a linear function of the environment RH. This is a reasonable assumption considering that the changes are driven by the RH and since the deformation field is small, namely, with an average of 1.5 voxels (26 nm) and a peak value of 5 voxels (87 nm). We found that a linear approximation proportional to the environmental RH, *RH*(*t_i_*), was sufficient to describe the time evolution of the deformation field. We then modeled the latter as

$$\Gamma \left(\overset{⃑}{r},t_{i}\right)=r_{H}\left(t_{i}\right)\Gamma \left(\overset{⃑}{r},t_{\text{end}}\right)$$ (4)

where *r_H_*(*t_i_*) is a linear ratio calculated from the environmental RH

$$r_{H}\left(t_{i}\right)=\frac{\text{RH}\left(t_{i}\right)-\text{RH}\left(t_{0}\right)}{\text{RH}\left(t_{\text{end}}\right)-\text{RH}\left(t_{0}\right)}$$ (5)

and where *t*_end_ = *t*_172_ is the starting time for the last sparse tomogram.

In this approximation, one 3D volume estimate can be reconstructed from the projections at each time frame

$$g_{i}\left(\overset{⃑}{r}\right)={\text{FBP}}_{\Gamma \left(\overset{⃑}{r},t_{i}\right)}\left\{P\left(t_{i}\right)\right\}$$ (6)

where $\Gamma^{\left(k\right)}\left(\overset{⃑}{r},t_{\text{end}}\right)$ denotes filtered back projection with a curved projection geometry given by the deformation field $\Gamma \left(\overset{⃑}{r},t\right)$ , using the adjunct matrix $A_{N}^{⊺}$ , as given by equation 3b in (*27*). Using these reconstructed volumes, the iterative update of the final deformation field $\Gamma \left(\overset{⃑}{r},t_{\text{end}}\right)$ can be described as

$$\begin{array}{c} \Gamma^{\left(k+1\right)}\left(\overset{⃑}{r},t_{\text{end}}\right)=\Gamma^{\left(k\right)}\left(\overset{⃑}{r},t_{\text{end}}\right)\\ +\sum_{i}\frac{r_{H}\left(t_{i}\right)}{\sum_{j}r_{H}\left(t_{j}\right)}\Delta \Gamma \left\{g_{i}\left(\overset{⃑}{r}\right),g_{0}\left(\overset{⃑}{r}\right)\right\} \end{array}$$ (7)

where ΔΓ is the update term based on the tomography reconstructions of the measured projections, given by equation 4 in (*27*). $\Gamma^{\left(k\right)}\left(\overset{⃑}{r},t_{\text{end}}\right)$ denotes the reconstructed deformation field in iteration *k*. Starting from an all-zero initial guess, five iterations were applied to the deformation field to get the final result, shown in Fig. 4C.

**Fig. 6.**
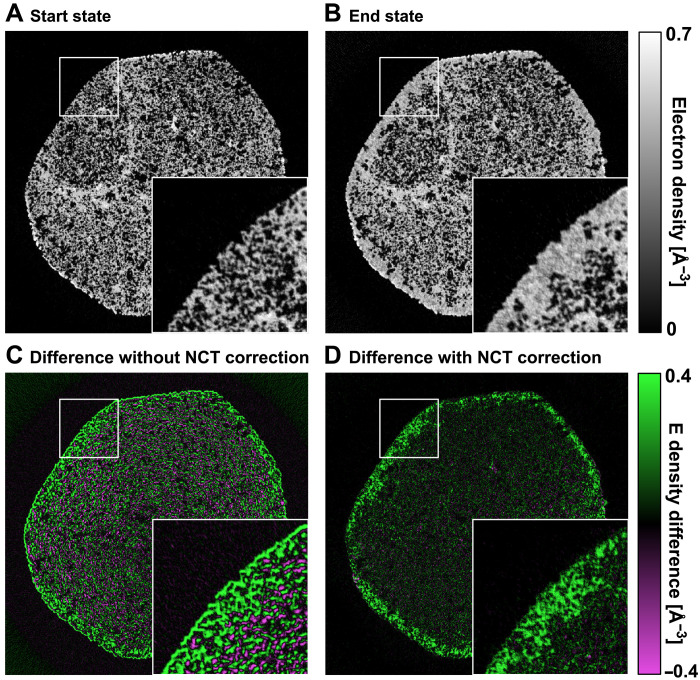
Demonstration of NCT correction. (**A**) An axial tomography slice from the reconstructed starting state of the sample. (**B**) Same tomography slice from the reconstructed end state of the sample. (**C**) Difference of ED between the start and end state of the sample without NCT correction, outlines around the pore edges are clearly visible, and the difference is heavily dominated by the sample geometrical expansion. (**D**) Difference between the two states after correction with NCT method, the outline artifacts are mostly removed, and the difference matches water filling of pores in the sample.

The improvement provided by the nonrigid tomography correction can be demonstrated by taking the difference of the tomography reconstructions from the first 20 sparse tomograms, which are measure at 81% RH in the first hour, and the last 20 sparse tomograms, which are measured at 92% RH in the last hour. Such reconstructed tomograms of the start state and the end state are shown in Fig. 6 (A and B, respectively). Figure 6 (C and D) shows the difference between the two states without and with the nonrigid correction, respectively. In Fig. 6C, we can see an outline contour around the sample and around individual pores, such edge artifacts are expected if the sample is expanding from its starting state during the dynamic process. In such case, the difference between the starting and end state is dominated by the geometrical deformation of the sample, and if not corrected, these artifacts are difficult to separate from water intake effects, because an increase of density at a voxel that starts empty can either be caused by water condensation or by nearby high-density material expanding into the voxel. This ambiguity affects the precision of further reconstruction of dynamics. For comparison, Fig. 6D shows the difference with the NCT correction, which accounts for the geometrical expansion of the sample. The corrected difference shows effects that are free of deformations and can be largely attributed to water intake and absorption, for example, showing empty voxels being filled with water. To note, the magnitude of the reconstructed deformation vector field, shown in Fig. 4C, correlates to the amount of water intake, shown in Fig. 6D. This correspondence indicates that certain proportion of sample deformation is related to, or induced by, water intake.

Similar to equation 4 in (*27*), a spatial convolution with a Gaussian filter, with a SD of 30 voxels, is applied to the calculation of the deformation vector field to regularize the result and reduce local variations. This step avoids abrupt changes of the deformation field at a small length scale, typically smaller than 20 pixels, i.e., 350 nm, which could result from overfitting noise and sparse sampling artifacts. Deformations at a smaller scale can be captured by the localized dynamics, as shown in Fig. 5 (E to H).

### Sparse dynamic tomography reconstruction

The core of the here presented methodology is the sparse parametrization and reconstruction of the local voxel-wise dynamics. Following the step function model described in Fig. 2, we can define the initial state, final state, and transition time for each voxel in the 3D volume as $N_{0}\left(\overset{⃑}{r}\right)$, $N_{1}\left(\overset{⃑}{r}\right)$ , and $T\left(\overset{⃑}{r}\right)$ , such that for any given time frame *t_s_*, the state of the sample is given by

$$N\left(\overset{⃑}{r},t_{s}\right)=\left\{\begin{array}{c} N_{0}\left(\overset{⃑}{r}\right),t_{s}\leq T\left(\overset{⃑}{r}\right)\\ N_{1}\left(\overset{⃑}{r}\right),t_{s}>T\left(\overset{⃑}{r}\right) \end{array}\right.$$ (8)

The reconstruction problem is then converted to retrieving $N_{0}\left(\overset{⃑}{r}\right)$ , $N_{1}\left(\overset{⃑}{r}\right)$ , and $T\left(\overset{⃑}{r}\right)$ . For this purpose, we developed an iterative refinement approach. The initial guess for the starting state, $N_{0}\left(\overset{⃑}{r}\right)$ , is

$$N_{0}\left(\overset{⃑}{r}\right)={\langle {\text{FBP}}_{\Gamma \left(\overset{⃑}{r},t\right)}\left\{P\left(t\right)\right\}\rangle }_{t_{0}\leq t\leq t_{20}}$$ (9)

where 〈 〉 denotes average over several time frames, in particular, here, the first 20 time frames are used. Similarly, for the initial guess of $N_{1}\left(\overset{⃑}{r}\right)$ , we used the average of the last 20 time frames, namely

$$N_{1}\left(\overset{⃑}{r}\right)={\langle {\text{FBP}}_{\Gamma \left(\overset{⃑}{r},t\right)}\left\{P\left(t\right)\right\}\rangle }_{t_{\text{end}-20}\leq t\leq t_{\text{end}}}$$ (10)

For the transition time $T\left(\overset{⃑}{r}\right)$ the initial guess are constant values

$$T\left(\overset{⃑}{r}\right)=\frac{t_{0}+t_{\text{end}}}{2}$$ (11)

**Fig. 7.**
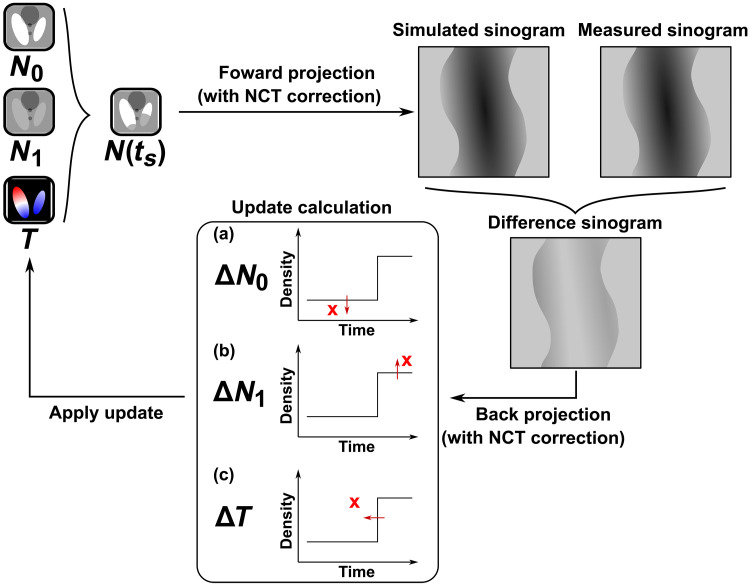
Illustration of the sparse dynamic reconstruction technique. Update strategy is illustrated by subfigures within the “update calculation” box. The step functions represent the current model, and the red crosses represent examples of reconstruction updates suggested by the back-projected correction $N_{c}\left(t_{s},\overset{⃑}{r}\right)$ . For illustration purposes, we portray corrections at three different values of *t_s_* in (a) to (c). The arrows represent the direction of refinement for different variables *N*_0_, *N*_1_, and *T* given by Eqs. 15 to 17.

Figure 7 shows a schematic of one iteration of the reconstruction process. In each iteration, one time frame *t_s_* between *t*_0_ and *t*_end_ is randomly selected. The modeled sample state at *t_s_* is then calculated on the basis of the current reconstruction with the step function model given in Eq. 8. Applying the conditional process to the whole sample volume then gives us the tomogram $N\left(\overset{⃑}{r},t_{s}\right)$ . From this modeled tomogram, we then apply a forward projection

$$\hat{P}\left(t_{s}\right)={\text{FP}}_{\Gamma \left(\overset{⃑}{r},t_{s}\right)}\left\{N\left(\overset{⃑}{r},t_{s}\right),\theta \left(t_{s}\right)\right\}$$ (12)

Here, ${\text{FP}}_{\Gamma \left(\overset{⃑}{r},t_{s}\right)}\left\{\right\}$ represents the projection operator under the curved projection geometry given by the deformation field $\Gamma \left(\overset{⃑}{r},t_{s}\right)$ , and θ(*t_s_*) denotes the sample orientation angles that were measured in the *s*th sparse tomogram.

We then compute the difference between the modeled and measured projections, followed by a back-projection of this difference to compute a 3D map of corrections, namely

$$N_{c}\left(t_{s},\overset{⃑}{r}\right)=R_{1}\left(\overset{⃑}{r}\right)*{\text{BP}}_{\Gamma \left(\overset{⃑}{r},t_{s}\right)}\left\{R_{2}\left(t_{s}\right)*\left[P\left(t_{s}\right)-\hat{P}\left(t_{s}\right)\right]\right\}$$ (13)

where ${\text{BP}}_{\Gamma \left(\overset{⃑}{r},t_{s}\right)}\left\{\right\}$ denotes the back projection with curved projection geometry given by the deformation field $\Gamma \left(\overset{⃑}{r},t_{s}\right)$ , *R*_1_ and *R*_2_ denote normalization arrays that are used in the simultaneous algebraic reconstruction technique (SART) (*54*, *55*), which can be calculated with unit-valued projections and a unit-valued tomogram, respectively.

$$\begin{array}{c} R_{1}\left(\overset{⃑}{r}\right)=\frac{1}{{\mathrm{BP}}_{\Gamma \left(\overset{⃑}{r},t_{s}\right)}\left\{P\left(t_{s}\right)=1\right\}},R_{2}\left(t_{s}\right)\\ =\frac{1}{{\mathrm{FP}}_{\Gamma \left(\overset{⃑}{r},t_{s}\right)}\left\{N\left(\overset{⃑}{r}\right)=1\right\}} \end{array}$$ (14)

The correction in Eq. 13 follows a strategy similar to SART to estimate the updates to the current reconstruction (*54*).

In the next step, we apply refinement corrections to $N_{0}\left(\overset{⃑}{r}\right)$ , $N_{1}\left(\overset{⃑}{r}\right)$ , and $T\left(\overset{⃑}{r}\right)$ based on values of $N_{c}\left(\overset{⃑}{r},t_{s}\right)$ . The correction values are given by

$$N_{0}\left(\overset{⃑}{r}\right)=N_{0}\left(\overset{⃑}{r}\right)+∆N_{0}\left(\overset{⃑}{r}\right)$$

$$∆N_{0}\left(\overset{⃑}{r}\right)=\left\{\begin{array}{c} \varepsilon *N_{c}\left(\overset{⃑}{r},t_{s}\right)\left[t_{s}\leq T\left(\overset{⃑}{r}\right)\right]\\ 0\left[t_{s}>T\left(\overset{⃑}{r}\right)\right] \end{array}\right.$$ (15)

$$N_{1}\left(\overset{⃑}{r}\right)=N_{1}\left(\overset{⃑}{r}\right)+∆N_{1}\left(\overset{⃑}{r}\right)$$

$$∆N_{1}\left(\overset{⃑}{r}\right)=\left\{\begin{array}{c} 0\left[t_{s}\leq T\left(\overset{⃑}{r}\right)\right]\\ \varepsilon *N_{c}\left(\overset{⃑}{r},t_{s}\right)\left[t_{s}>T\left(\overset{⃑}{r}\right)\right] \end{array}\right.$$ (16)

$$T\left(\overset{⃑}{r}\right)=T\left(\overset{⃑}{r}\right)+∆T\left(\overset{⃑}{r}\right)$$

$$∆T\left(\overset{⃑}{r}\right)=\left\{ \begin{array}{c} 0,t_{s}\leq T\left(\overset{⃑}{r}\right)\&\text{sign}\left[N_{1}\left(\overset{⃑}{r}\right)-N_{0}\left(\overset{⃑}{r}\right)\right]\text{*}N_{c}\left(t_{s},\overset{⃑}{r}\right)\leq 0\\ -\varepsilon^{*}\tau^{*}\delta \left(t_{s},\overset{⃑}{r}\right)*N_{c}\left(\overset{⃑}{r},t_{s}\right),t_{s}\leq T\left(\overset{⃑}{r}\right)\&\text{sign}\left[N_{1}\left(\overset{⃑}{r}\right)-N_{0}\left(\overset{⃑}{r}\right)\right]\text{*}N_{c}\left(t_{s},\overset{⃑}{r}\right)>0\\ -\varepsilon^{*}\tau^{*}\delta \left(t_{s},\overset{⃑}{r}\right)*N_{c}\left(\overset{⃑}{r},t_{s}\right),t_{s}>T\left(\overset{⃑}{r}\right)\&\text{sign}\left[N_{1}\left(\overset{⃑}{r}\right)-N_{0}\left(\overset{⃑}{r}\right)\right]\text{*}N_{c}\left(t_{s},\overset{⃑}{r}\right)<0\\ 0,t_{s}>T\left(\overset{⃑}{r}\right)\&\text{sign}\left[N_{1}\left(\overset{⃑}{r}\right)-N_{0}\left(\overset{⃑}{r}\right)\right]\text{*}N_{c}\left(t_{s},\overset{⃑}{r}\right)\geq 0 \end{array}\right.$$ (17)

where ε is an update relaxation factor that is gradually reduced during iterations for convergence, we typically use

$$\varepsilon =10^{-4}*0.998^{k},k=1,2,\ldots$$ (18)

where *k* is the iteration number, and τ is a scaling ratio based on the units used for time and tomogram values. It can be estimated using quantitative values of *T* and *N*_0_

$$\tau =\frac{\langle T\left(\overset{⃑}{r}\right)\rangle }{\langle N_{0}\left(\overset{⃑}{r}\right)\rangle }$$ (19)

where 〈 〉 denotes average over the whole volume, and $\delta \left(t_{s},\overset{⃑}{r}\right)$ is a time relaxation ratio given by

$$\delta \left(t_{s},\overset{⃑}{r}\right)=\frac{1}{8}\frac{\left(t_{\text{end}}-t_{0}\right)}{\left(t_{\text{end}}-t_{0}\right)+8|T\left(\overset{⃑}{r}\right)-t_{s}|}$$ (20)

which reduces the correction if the transition time of the target voxel is far from the current time frame *t_s_*. Adding this relaxation ratio is important for dealing with noise in the data and reduce changes of the correction overshooting.

Equations 15 and 16 are used to calculate the updates applied to the start and end state tomograms, respectively. These updates are similar to the conventional SART method, but with changes constrained to the voxels that apply to each of them at the time frame *t_s_*. As illustrated in Fig. 7 (a and b), for voxels of which $T\left(\overset{⃑}{r}\right)$ is smaller than *t_s_*, the update is applied to the start state and conversely, to the end state for voxels of which $T\left(\overset{⃑}{r}\right)$ is larger than *t_s_*.

As illustrated in Fig. 7c, the refinement of transition time in Eq. 17 is applied on the basis of the sign and amplitude of the correction, $N_{c}\left(t_{s},\overset{⃑}{r}\right)$ , and the state of the voxel at time *t_s_*. In the example illustrated in Fig. 7c, we have $t_{s}<T\left(\overset{⃑}{r}\right)$, $\text{sign}\left[N_{1}\left(\overset{⃑}{r}\right)-N_{0}\left(\overset{⃑}{r}\right)\right]>0$ , and $N_{c}\left(t_{s},\overset{⃑}{r}\right)>0$ , and for this case, the correction ∆*T* is in the negative direction, since the error would be potentially reduced if the value of *T* of that particular voxel was reduced. This situation corresponds to the second line of Eq. 17. When iterated over all the time frames, the transition time of all voxels will converge at the time frame where the change occurred.

After one iteration of refinement, another time frame *t_s_* is selected randomly and the whole process is iterated until convergence. In our case, we applied 2500 iterations for the numerical simulations and 5000 iterations for the experimental data, which took 5 min and 8 hours, respectively, on our computation node powered by an NVIDIA Tesla V100 GPU.

### Spatial resolution estimate

To estimate the spatial resolution of the reconstruction, the measured data were split into two subsets by taking every second time frame, namely, the first set containing projections {*P*(*t*_0_), *P*(*t*_2_), ⋯, *P*(*t*_172_)}, and the second set containing {*P*(*t*_1_), *P*(*t*_3_), ⋯, *P*(*t*_173_)}. The nonrigid correction and sparse dynamic tomography reconstruction method was then applied to both sets independently, to reconstruct two separate sets of results, namely, $\{N_{0}^{\left(1\right)}\left(\overset{⃑}{r}\right)$ , $N_{1}^{\left(1\right)}\left(\overset{⃑}{r}\right)$ , and $T^{\left(1\right)}\left(\overset{⃑}{r}\right)\}$ and $\{N_{0}^{\left(2\right)}\left(\overset{⃑}{r}\right)$ , $N_{1}^{\left(2\right)}\left(\overset{⃑}{r}\right)$ , and $T^{\left(2\right)}\left(\overset{⃑}{r}\right)\}$.

**Fig. 8.**
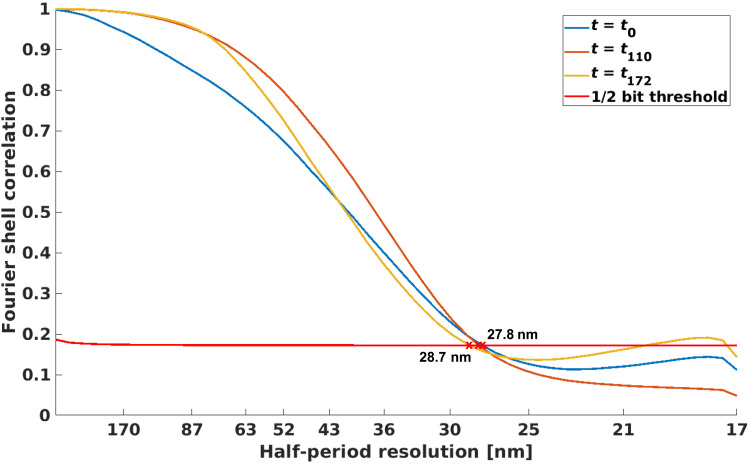
Fourier shell correlation. Different curves represent FSCs between reconstructions from two independent subsets of the data at different time frames. Half-period spatial resolution estimates between 27.8 and 28.7 nm are given by their first intersection with the 1/2 bit threshold curve.

With these two sets of reconstructed results, at each time frame *t_i_*, we calculate the reconstructed sample volume as given by Eq. 8, namely, $N^{\left(1\right)}\left(\overset{⃑}{r},t_{i}\right)$ and $N^{\left(2\right)}\left(\overset{⃑}{r},t_{i}\right)$ . Then, we compute the Fourier shell correlation (FSC) (*56*) between the two reconstructed volumes and compare the correlation curve to the 1/2 bit threshold, as shown in Fig. 8. The estimate of spatial resolution is given by the coordinate of the first intersection. For all reconstructed time frames in the process, the half-period spatial resolution was estimated to be in the range of 27.8 to 28.7 nm. The correlation curves of a few example time frames are shown in Fig. 8. To note, these resolution values are comparable to typical PXCT imaging results measured on the same instrument in static condition and without sparse sampling (*57*, *58*).

### Numerical simulations

For demonstration and characterization of the sparse dynamic reconstruction method, we carried out numerical simulations and reconstructions. In this manner, we could study, for example, the effects of noise and other mismatches between the dynamics and the models used for reconstruction.

**Fig. 9.**
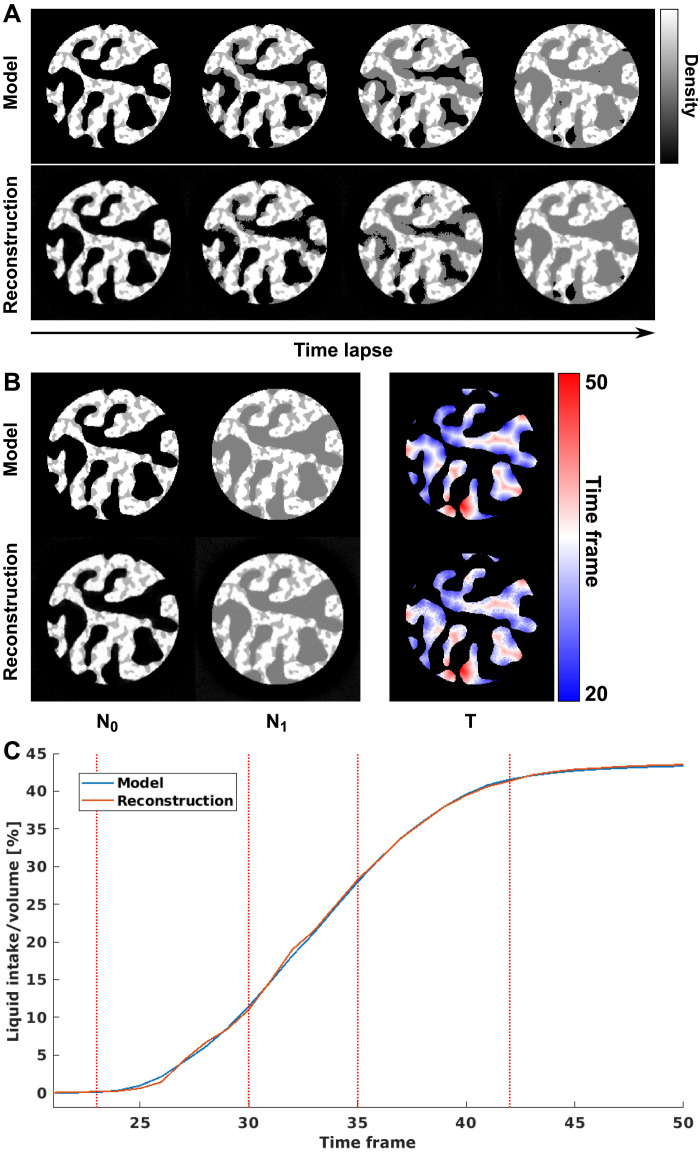
Numerical simulation of liquid filling process in a porous sample. (**A**) Model and reconstructed time-lapse tomograms of the dynamic process. (**B**) Model and reconstructed *N*_0_, *N*_1_, and *T*. (**C**) Comparison of total amount of liquid intake over time calculated from the model and the reconstruction. Dashed lines refer to the time frames shown in (A).

For the first scenario, we simulate a liquid filling process in a two-phase porous material. The model is generated with a pillar shape within an array of 200 × 200 × 5 voxels, and the pores are filled with simulated liquid starting from the pore surfaces to the center, as shown in the top row of Fig. 9A. The diameter of the pillar is 160 voxels, which means that the number of projections needed to satisfy the Crowther criterion is 250. Within each time frame, six projections were simulated at different sample rotation angles, which correspond to a sparsity ratio of 2.4%. A total of 80 time frames were simulated, the liquid filling starts at the 20th time frame and finishes at the 50th frame.

The reconstructed dynamic process from these projections is shown in the bottom row of Fig. 9A. The results agree quite well with the ground truth, both on the filling time and shape of the liquid-filling front. Quantitative comparison of the total amount of liquid intake, shown in Fig. 9C, also shows good agreement between the model and reconstruction. Figure 9B shows the values of *N*_0_, *N*_1_, and *T*, which uniquely define the dynamic process, and top and bottom panels show the model and the reconstruction, respectively. The root mean square error (RMSE) for the transition time *T* was 1.38 time frames, which indicates a relatively accurate reconstruction of the dynamic process with 30 time frames duration.

The second simulation is intended to evaluate the robustness of the reconstruction method against noise, in particular to understand the effect of sparse sampling and compare directly to conventional sampling that satisfies the Crowther criterion. The liquid-filling simulation was repeated with different sparsity-sampling ratios, ranging from 1 to 50%. Random noise of Gaussian distribution with 2% SD of the average value of the projections was added to the projections to simulate measurement noise. The error on the reconstruction was estimated using the normalized RMSE, given by

$$e=\sqrt{\frac{\sum_{t}\sum_{\overset{⃑}{r}}{\left[N\left(\overset{⃑}{r},t\right)-N_{m}\left(\overset{⃑}{r},t\right)\right]}^{2}}{\sum_{t}\sum_{\overset{⃑}{r}}N_{m}{\left(\overset{⃑}{r},t\right)}^{2}}}$$ (21)

where $N\left(\overset{⃑}{r},t\right)$ denotes the reconstructed tomograms at each time frame, and $N_{m}\left(\overset{⃑}{r},t\right)$ denotes model tomograms or “ground truth” at each time frame.

The NRMSE versus sparsity ratio is shown in fig. S2. The results were compared with the reference value of conventional filtered back-projection (FBP) reconstructions with the same amount of noise added per projection and with full angular sampling, which corresponds to 250 projections per time frame. As expected, the error drops with higher sparsity ratio and even outperforms the conventional FBP method at >20% ratio. This is due to the iterative refinement and the fact that we use in our reconstruction a large number of projections, i.e., all time frames simultaneously. In the range of 3 to 15% sparsity ratio, the error is relatively constant, and it grows more sharply when the ratio goes below 3%. Notice that two sample states and one transition time, namely, *N*_0_, *N*_1_, and *T*, are to be reconstructed from 80 sparsely sampled tomograms at different time frames. Taking into account the correlations between these variables, this gives a rough estimate of lower-bound ratio of 2.5/80 or 3.12% sparsity ratio, to have enough total projections for the reconstruction. The estimate agrees with the observed sharper increase of the error below 3% sparsity ratio.

**Fig. 10.**
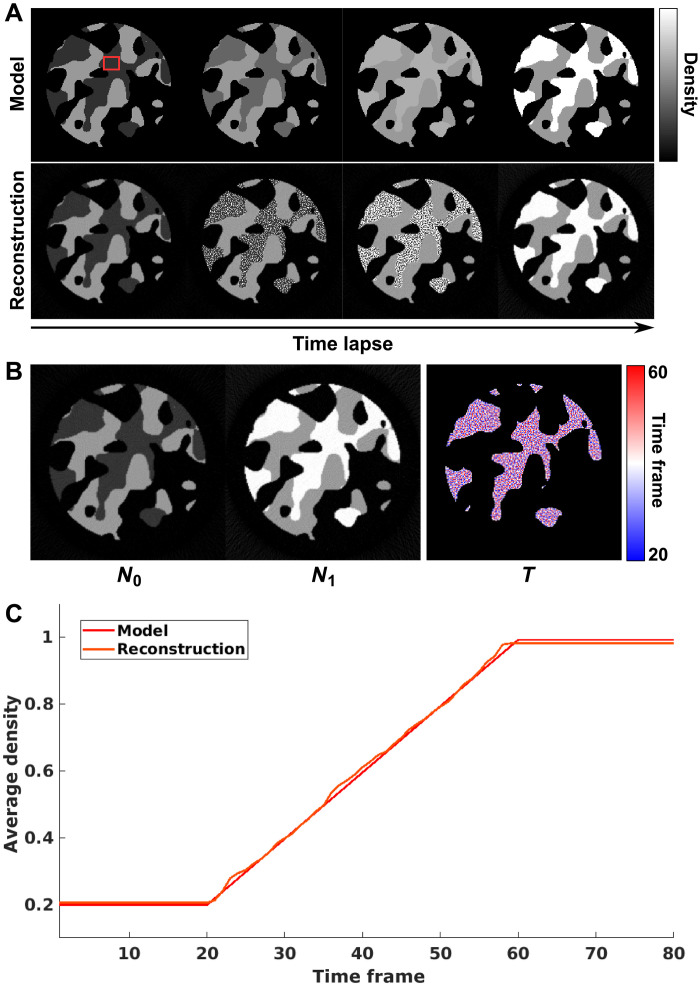
Numerical simulation of gradual linear local density transition. (**A**) Model and reconstructed time-lapse tomograms of the dynamic process based on gradual linear density changes. (**B**) Reconstructed results of *N*_0_, *N*_1_, and *T*. (**C**) Comparison between model and reconstructed average density dynamics of a 20 × 15 voxel region marked by red rectangle in (A).

Another important aspect to consider is the performance of the reconstruction code when the assumption of a single step function change per voxel is not satisfied. For this purpose, we studied the third numerical simulation of gradual linear transition of material density. Again, a pillar of porous material was generated with 160 voxels diameter within an array of 200 × 200 × 5 voxels. In the dynamic process, the density of part of the structure is increased linearly over 40 time frames, within a total period of the simulation of 80 time frames. The same sparsity ratio of 2.4% was applied, with six projections simulated at each time frame. The model and reconstructed results are shown in Fig. 10A.

The dynamics per voxel are modeled in the reconstruction with step function, which does not allow representation of a gradual change. The reconstruction algorithm resolves this by splitting the temporal transition into several voxels, such that each voxel has a step function, but the average of a few voxels undergoes a more gradual transition. In the reconstruction in Fig. 10B, this can be observed as noise that resembles a salt-and-pepper pattern. In effect, the algorithm creates a compromise of the spatial resolution of the dynamics to accommodate a more complex temporal behavior. In Fig. 10A, one can also see that the changing voxels were correctly identified, so the spatial locations of the transition are recovered correctly.

The value for the average density of a region of 20 × 15 voxels, indicated by a red square in Fig. 10A, is shown in Fig. 10C. The average shows a relatively good match with the model, indicating that the method still provides useful information for these cases. In Fig. 10C, small differences of the average values for the start and the end states can be observed. These differences are potentially due to the step function model not being able to capture the small changes at the very start and very end of the transition, resulting in a slight delay of the reconstructed start time of transition and conversely, an earlier end time for the end of the dynamics. This mismatch in the reconstruction of the exact start and end of the dynamics also causes a small bias in the reconstructed densities of *N*_0_ and *N*_1_. While these effects can be removed by introducing a more complex function for the voxel-level response, they can alternatively be alleviated by upsampling the reconstruction voxels. With the latter strategy, a resolution element in the reconstruction contains several voxels, which can be leveraged by the algorithm to represent more complex dynamics. It should be noted that in our experimental demonstration, we applied this spatial oversampling strategy by using a reconstruction voxel size of 17.4 nm, while the imaging resolution is about 28 nm.
