# Supplementary material for: Dynamic sparse x-ray nanotomography reveals ionomer hydration mechanism in polymer electrolyte fuel-cell catalyst
Source: Sci Adv. 2024 Oct 9;10(41):eadp3346. doi: 10.1126/sciadv.adp3346 (PMC11463282; doi:10.1126/sciadv.adp3346)
Supplement: Supplementary file 1 — Figs. S1 and S2 [file sciadv.adp3346_sm.pdf]

Science Advances
AAAS

Supplementary Materials for

Dynamic sparse x-ray nanotomography reveals ionomer hydration mechanism in polymer electrolyte fuel-cell catalyst

Zirui Gao *et al.*

Corresponding author: Zirui Gao, zirui.gao@psi.ch; Johannes Ihli, Johannes.Ihli@materials.ox.ac.uk; Manuel Guizar-Sicairos, manuel.guizar-sicairos@psi.ch

Sci. Adv. **10**, eadp3346 (2024)
DOI: 10.1126/sciadv.adp3346

This PDF file includes:

Figs. S1 and S2

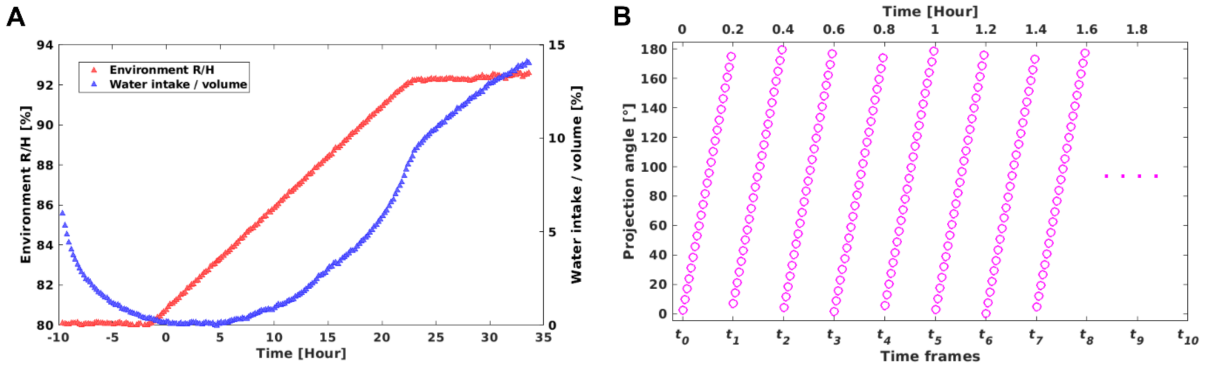

Figure S1. Acquisition for the dynamic hydration process.

A. The full measurement process, including 10 hours wait time for the sample to stabilize after initial tests of the humidity environmental chamber. Each triangle represents the starting time of one sparsely sampled tomogram, with relative humidity values shown in blue and the amount of water intake shown in red. The latter is estimated using the integrated electron density obtained from 2D projections. **B.** Projection angles measured in each sparse tomogram.

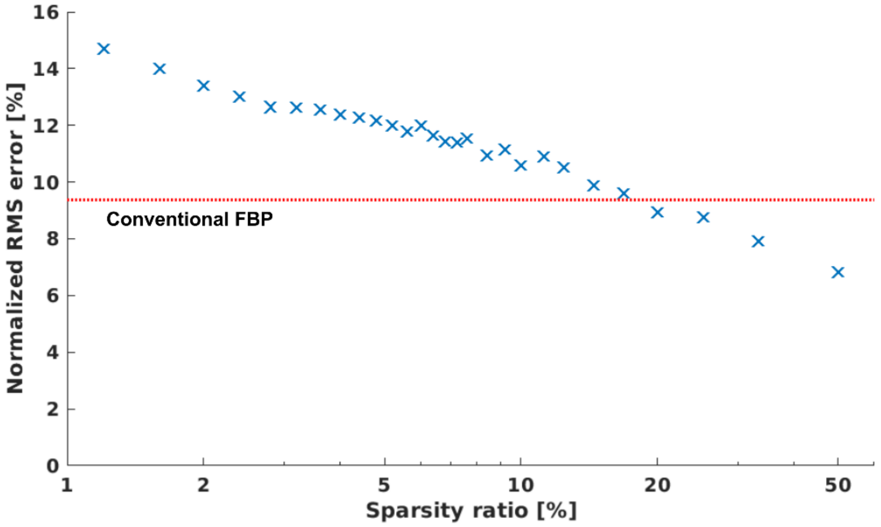

Figure S2. Noise level of the reconstructions at different sparsity ratios.

Normalized RMS errors (NRMSE) between the reconstruction and the model, or ‘ground truth’, for different sparse sampling ratios are shown. Dashed line represents the NRMSE of the filtered back-projection (FBP) method with conventional sampling, i.e. sampled following the Crowther criterion without sparsity, as reference.
